# SITTING PATTERNS, PHYSICAL ACTIVITY, AND PHYSICAL FUNCTIONING IN OLDER ADULTS

**DOI:** 10.1093/geroni/igz038.072

**Published:** 2019-11-08

**Authors:** Dori E Rosenberg, Mikael Anne Greenwood-Hickman, Rod walker, KatieRose Richmire, Paul Crane, Eric Larson, Andrea LaCroix

**Affiliations:** 1 Kaiser Permanente Washington Health Research Institute, Seattle, Washington, United States; 2 KPWHRI, seattle, Washington, United States; 3 UW, Seattle, Washington, United States; 4 UC San Diego, La Jolla, Washington, United States

## Abstract

We examined cross-sectional associations between physical function and device-based (activPAL) sedentary patterns and physical activity. Physical function tasks included time to complete 5 chair stands and walk a 10-foot gait speed course. We estimated associations using linear regression models adjusting for age and sex; coefficients represent estimated change in mean activPAL measures associated with each second increase in gait/chair stands time. Longer gait speed times were associated with more total sitting time (b=0.19, p < 0.01), fewer steps (b=-788.0, p<0.001), fewer sitting breaks (b=-1.7, p<0.01), and more prolonged sitting bouts (b=0.19, p<0.01). Longer chair stand times were associated with more total sitting time (b=0.06, p<0.001), less standing time (b=-0.04, p<0.01), fewer steps (b=-176.8, p<0.001), fewer sitting breaks (b=-0.45, p<0.01), and more prolonged sitting bouts (b=0.07, p<0.001). Prolonged patterns of sitting time and higher total sitting time, in addition to lower physical activity, were consistently associated with worse physical function.

